# Characterization of Apps and Other e-Tools for Medication Use: Insights Into Possible Benefits and Risks

**DOI:** 10.2196/mhealth.4149

**Published:** 2016-04-06

**Authors:** Linda Wilhelmina Maria van Kerkhof, Catharina Walthera Egbertha van der Laar, Charlie de Jong, Marjolein Weda, Ingrid Hegger

**Affiliations:** ^1^ National Institute for Public Health and the Environment (RIVM) Centre for Health Protection Bilthoven Netherlands

**Keywords:** mobile apps, drugs, eHealth, mHealth, medication use

## Abstract

**Background:**

In the past years, an enormous increase in the number of available health-related applications (apps) has occurred, from approximately 5800 in 2011 to over 23,000 in 2013, in the iTunes store. However, little is still known regarding the use, possible effectiveness, and risks of these applications. In this study, we focused on apps and other e-tools related to medicine use. A large subset of the general population uses medicines and might benefit from tools that aid in the use of medicine.

**Objective:**

The aim of the present study was to gain more insight into the characteristics, possible risks, and possible benefits of health apps and e-tools related to medication use.

**Methods:**

We first made an inventory of apps and other e-tools for medication use (n=116). Tools were coded by two independent researchers, based on the information available in the app stores and websites. Subsequently, for one type of often downloaded apps (aimed at people with diabetes), we investigated users’ experiences using an online questionnaire.

**Results:**

Results of the inventory show that many apps for medication use are available and that they mainly offer simple functionalities. In line with this, the most experienced benefit by users of apps for regulating blood glucose levels in the online questionnaire was “information quick and conveniently available”. Other often experienced benefits were improving health and self-reliance. Results of the inventory show that a minority of the apps for medication use has potentially high risks and for many of the apps it is unclear whether and how personal data are stored. In contrast, online questionnaire among users of apps for blood glucose regulation indicates that they hardly ever experience problems or doubts considering reliability and/or privacy. Although, respondents do mention to experience disadvantages of use due to incomplete apps and apps with poor ease of use. Respondents not using app(s) indicate that they might use them in the future if reliability of the apps and instructions on how to use them are more clear.

**Conclusions:**

This study shows that for apps and e-tools related to medicine use a small subset of tools might involve relatively high risks. For the large group of nonmedical devices apps, risks are lower, but risks lie in the enormous availability and low levels of regulation. In addition, both users and nonusers indicated that overall quality of apps (ease of use, completeness, good functionalities) is an issue. Considering that important benefits (eg, improving health and self-reliance) are experienced by many of the respondents using apps for regulating blood glucose levels, improving reliability and quality of apps is likely to have many profits. In addition, creating better awareness regarding the existence and how to use apps will likely improve proper use by more people, enhancing the profits of these tools.

## Introduction

In the past years, there has been an enormous growth in the availability of health-related applications for mobile devices, so-called “health apps” and Web-based tools, here called “health apps and e-tools”. Apps are mainly used on mobile devices and are available in app stores (such as the iTunes store or Google Play), whereas Web-based e-tools are mainly designed for non-mobile devices (such as Internet applications for PC). The health apps and e-tools are designed for patients and for use by health care professionals to aid them in their daily practice.

The number of available health apps has increased dramatically from roughly 5800 in January 2011 [1], to 13,000 in August 2012, to over 23,000 health care apps in June 2013, in the United States iTunes store alone, of which 16,275 apps were for consumers [2]. Apart from the enormous increase in the availability of apps, use of health apps and e-tools is encouraged in the process to increase patient empowerment and patient participation in health care [3,4-6].

The massive increase in health apps has drawn attention of regulatory authorities, because of possible risks associated with them. Regulatory authorities have in particular concerns about apps that turn a mobile device into a medical device and, as such, need to be regulated [7,8]. Recently, the US Food and Drug Administration (FDA) has published guidelines on their control of health care apps [8]. This is followed by increased attention for health care apps by other regulatory authorities, such as the Dutch Healthcare Inspectorate [9]. In addition, numerous media have paid attention to the growing number of health e-tools and possible risks [10,11-13].

The FDA has announced to focus its regulatory actions on “higher risk mobile apps” [8,14] which mainly consist of apps that require an attachment to the phone, to enable measurement, diagnosis or treatment of a medical problem. Examples of these are apps and devices that enable a mobile phone to monitor heart function or produce tones for audiometry [14]. However, especially the availability and use of “lower risk mobile medical apps” as well as apps that are “nonmedical devices”, which are not designated to convert a mobile phone into a medical device, are booming. Several previous studies have indicated that the overall quality of medical apps is poor, for example regarding accuracy, clinical usability, scientific evidence for effectiveness, adherence to guidelines, expert involvement during development and reliability [6,15-19].

In the present study, we focus on health apps and e-tools related to medication use for patients and/or health care professionals. We defined medication use as any use of licensed, prescribed or nonprescribed medicinal products by a patient, an informal caregiver or health care professional to treat a disorder or relieve symptoms of the patient. A large subset of the general population uses prescribed medicine (68% in the United States [20]; 37% in the Netherlands [21]); hence, they are the target market of a substantial subset of these tools. Importantly, they also represent a possible group of users in which risks associated with the tools might occur more easily. For example, monitoring your body weight (general health app) or monitoring your glucose levels for insulin administration (health app related to medication use) are likely associated with different risks.

The aim of the present study was to gain more insight into the characteristics, possible risks, and possible benefits of health apps and e-tools related to medication use. For this purpose, first an inventory of apps and e-tools for medication use was made. After assessment of characteristics and potential risks and benefits, one target group of often downloaded apps was selected (persons with diabetes). Using an online questionnaire, the use of apps for regulating blood glucose levels, characteristics, and experienced benefits and risks by persons with diabetes were investigated.

## Methods

### Search Strategy Inventory of Apps

The search strategy focused on apps and e-tools available in the Netherlands, and therefore, we used Dutch search terms. However, we included apps and e-tools available in Dutch or English, since we expected that English apps will be used by a substantial amount of Dutch users as well. To identify apps related to medication use, we performed searches in the Google Play store and Apple iTunes store. In the app stores, the following search terms were used: “medicine”, “drugs”, “diabetes”, “asthma”, “breast cancer”, “prostate cancer”, “cardiovascular diseases”, “ADHD” and a combination of the terms “medicine” and “diabetes” (the Dutch terms can be found in Multimedia Appendix 1 supplementary data A). The terms for diseases were chosen, since these diseases are known to involve medication use, have self-management aspects, and resemble a broad spectrum of diseases resulting in a wide range of functionalities of the apps. For all terms, we included the first 20 hits. For the general terms “medicine” and “drugs”, the first 50 hits were included.

In addition, to identify other e-tools, we searched several Dutch websites (n=11) using Google (for details, please refer to Multimedia Appendix 1 supplementary data A). We selected websites that publish news on eHealth technologies, are publically available, and represent different modalities of care. We searched all websites using the following search string: eHealth OR medicine OR “online tool” OR “Web-based medicine” OR “ICT and health care” OR telemedicine OR tele-monitoring OR app. In addition, depending on the website, we used only parts of this search string to avoid large amounts of irrelevant hits (eg, the term “eHealth” in a website focused on eHealth will render numerous irrelevant results, while the same term in a website for pharmacists is very useful). We performed the searches between June and October 2013.

### Selection of Relevant Apps and Other e-Tools

The app and e-tool searches yielded a large number of results (app stores total: 314 apps; 217 Google Play Store and 97 from the iTunes store; e-tools: 269 messages from 11 websites). In order to filter out the relevant apps and e-tools, two independent researchers made a selection using the following criteria: (1) being an app or e-tool (defined as a tool used on an electronic device that requires some form of input, hence, this excludes regular Internet sites); (2) clearly relating to use of medication in humans; (3) excluding apps related to alternative medicines;

(4) being available in Dutch or English, based on available app print screen or description.

### Coding of Apps and Other e-Tools

We coded the selected apps and e-tools in ATLAS.ti (version 7.1.3), based on a coding scheme consisting of 16 questions about the characteristics and possible risks and benefits of each app or e-tool (see Multimedia Appendix 1 supplementary data B). Two separate researchers coded all e-tools; afterwards, we discussed discrepancies in coding until a consensus was reached. Codes were based on the information provided by the developers of the tools (information found in the ITunes Store, Google Play Store or on the Internet in case of Internet e-tools). No apps were downloaded and none of the tools were actually used.

The coding scheme was established using literature [18,22] and pilot codes. The codes are divided into three categories. The first category includes codes related to the main characteristics, which were: (1) intended goal(s), (2) intended user(s), (3) type of tool (eg, app, Internet, etc), and (4) number of downloads in the Google Play store. Unfortunately, no download data were available for the apps that were found in the ITunes Store and were thus not coded in terms of number of downloads. In the second category, we coded different aspects that are generally named as benefits of eHealth, such as lowering health costs, improving health care, enhancing patient self-management and self-reliance [23-25]. In the third category, we included codes related to the possible risks of the tools. This involved aspects such as possible (user) data upload, the absence of health care professionals when obtaining or using the tool, and the possibility of health-related harm after using the e-tool. In addition, all the tools were checked to see if they could be considered as medical devices under EU law. To properly classify and code them, the Medical Devices Guidance Document on Qualification and Classification of stand-alone software was used [7]. Classification of medical devices is based on the intended use of the developer/producer and we assessed the “intended use” on the basis of the information available in the app stores and on the Internet. If e-tools could be classified as type II medical devices, they were also coded for whether or not they could be accessed by non-health care professionals, ie, patients. For additional information on the coding criteria, see Multimedia Appendix 1 supplementary data B.

### Online Questionnaire

We investigated use of apps by people with diabetes. This user group was chosen based on the finding that these types of apps are often downloaded (see Results section). Questionnaires were developed in collaboration with the Dutch Diabetes Association (DVN) as part of a larger survey into app use by persons with diabetes. The DVN has about 49,000 members, which represent 6 % of the people with diabetes in the Netherlands [26]. Questions were aimed at providing insight into use of apps for blood glucose levels, types of apps used, and experienced benefits and risks. Focus on apps for blood glucose levels were chosen since these apps are related to medication use and some of these apps might have higher risks (see Results-Inventory of Apps for Medication Use). Questions were based on the inventory codes. For overview of the questions see Multimedia Appendix 1 supplementary data C. Questback software (Oslo, Norway) was used. DVN members were invited to participate via the DVN newsletter and website. Participation was completely voluntary, anonymous, and no compensation in any form was provided for participation. The questionnaire was open for respondents for 6 weeks in May and June 2015. Participants could check their answers by going back in the questionnaire. No preventive measures were taken to avoid participants completing the questionnaire multiple times, since this is highly unlikely with the type of questions in the questionnaire (no incentive to do so for participants).

## Results

### Inventory of Apps and e-Tools for Medication Use

#### Main Characteristics

**Figure 1:**
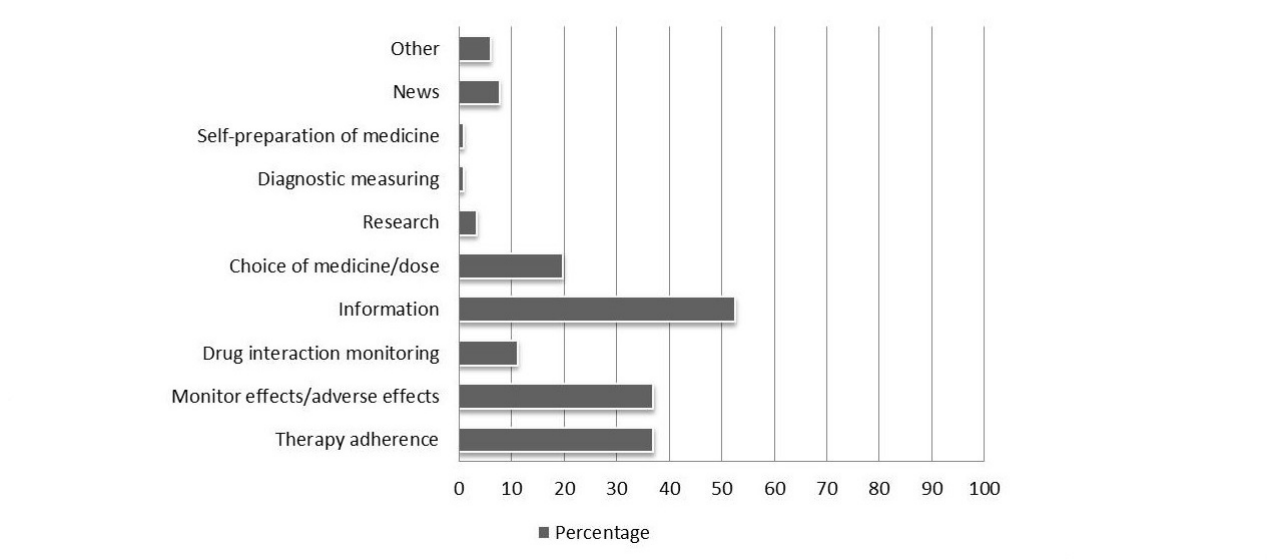
Functionalities of the tools. Data is presented as percentage of total number of tools (n = 116). Tools can have multiple functionalities.

After carefully going through all the search results, 116 tools were selected as relevant for the research project by using the aforementioned selection criteria. The three most frequent (intended) functions of the tools were providing users with information/education (52.6%, 61/116), assisting users with their therapy adherence (37.1%, 43/116), and helping users monitor the effect and possible side effects of their medication (37.1%, 43/116). Less common (intended) functions included helping users choose a medication or dose (19.8%, 23/116), drug interaction monitoring (11.2%, 13/116), and providing users with news (7.7%, 9/116) (Figure 1 and Multimedia Appendix 1 supplementary data D Table D1). For examples of the apps and their functionalities, see Multimedia Appendix 1 supplementary data D Fig D1.

The majority of the selected tools were apps (87.1%, 101/116). Only 12.1% (14/116) consisted of Internet e-tools and 5.2% (6/116) could be classified as a measurement device.

**Figure 2:**
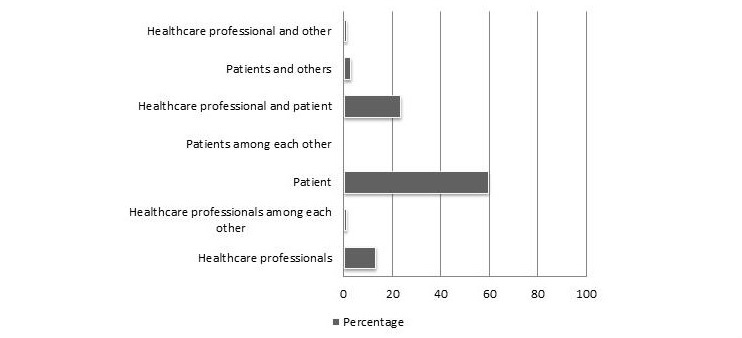
Intended users of the tools. Data is presented as percentage of total number of tools (n=116). Tools can have multiple intended users.

In terms of intended users, the main target seemed to be patients with 59.5% (69/116) of the selected tools meant for this group. The second largest group of tools (23.3%, 27/116) aimed at the interaction between patients and health care professionals. Only 12.9% (15/116) of tools were solely targeting health care professionals. For complete overview of results see Figure 2 and Multimedia Appendix 1 supplementary data D Table D1.

**Figure 3:**
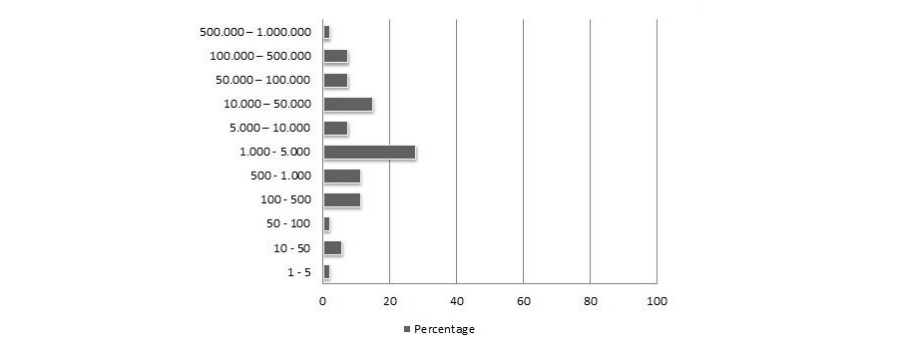
Frequency distribution of number of downloaded (for apps from the Google Play store). Data is presented as percentage of total number of apps (n=54).

We observed that the majority of the selected apps were downloaded sparsely; 59% (32/54) was downloaded less than 5000 times worldwide (Figure 3). The most frequently download category was 1000–5000 downloads per app (28%, 15/54). Only a few apps (4%, 2/54) were downloaded more than 500,000 times. It is important to note that the numbers of downloads do not necessarily equal or represent the actual usage.

#### Possible Benefits

To investigate possible benefits of eHealth tools, we selected different aspects that are generally named as benefits of eHealth, such as lowering health costs, improving health care, enhancing patient self-management, and self-reliance [23-25]. For criteria of the codes, see Multimedia Appendix 1 supplementary data B.

First, we assessed whether the tool could potentially contribute to an improvement in the user’s health. This was the case in 65.5% (76/116) of the selected tools; 33.6% (39/116) of the tools did not have this potential. Second, more than half of the tools could potentially enhance a patient’s self-reliance (52.6%, 61/116), while 31.9% (37/116) could not. Third, we assessed whether the selected tools had the potential to lower health care costs. More than half of them (55.2%, 64/116) had this potential while 40.5% (47/116) did not. Fourth, the tools’ potential to contribute to a patient’s self-management was assessed. This possible benefit was found less frequently among the selected tools than the other examined benefits and was only found in 35.3% (41/116) of the e-tools. For an overview of these results, see Table 1.

**Table 1:** Table . Assessment of possible benefits of apps and other e-tools for medication use; data is presented as percentage of total number of tools investigated (N=116).

Coding	Yes	No	Not assessable	Not applicable
	n (%)	n (%)	n (%)	n (%)
Potentially improves health	76 (65.5)	39 (33.6)	1 (0.9)	0 (0)
Potentially enhances self-reliance	61 (52.6)	37 (31.9)	2 (1.7)	16 (13.8)
Potentially lowers health care costs	64 (55.2)	47 (40.5)	5 (4.3)	0 (0)
Potentially contributes to self-management	41 (35.3)	56 (48.3)	3 (2.6)	16 (13.8)

#### Possible Risks

We coded eight possible risks of the e-tools: (1) classification as medical device, (2) data upload (privacy), (3) involvement of health care professional in obtaining and (4) use of the tool, (5) accessibility to non-health care professionals, (6) replacement of health care professional, (7) risks of erroneous use, and (8) risks of erroneous design. These possible risks are further described below. For a complete overview of criteria of these codes, see Multimedia Appendix 1 supplementary data B.

For all tools, we assessed if they could be classified as a medical device according to the EU regulations. To properly classify the tools, the Medical Devices Guidance Document on Qualification and Classification of stand-alone software was used [7]. We classified a small number of tools as a medical device (13.8%, 16/116), which were all classified as a type II medical device. The majority of tools, 86.2% (100/116), was not classified as medical devices. For none of the apps classified as a medical device, a CE mark was observed in the app store; however, since apps were not downloaded it is possible that CE mark was present in the app itself. If we classified a tool as a type II medical device, and if health care professionals were the intended user group, we also examined whether they were accessible to non-health care professionals. Only eight tools satisfied both criteria. Out of these, seven were accessible to non-health care professionals and for one app, these coding could not reliably be assessed. Thus, the majority of type II medical device tools designed for use by health care professionals were accessible to the general public. In summary, these results indicate that most apps and other e-tools related to medication use represent nonmedical devices. However, there are several type II medical devices identified with potentially high risks. In addition, the limited number of type II medical device apps designed for health care professionals are accessible to the general public.

The occurrence of data upload holds a possible risk related to privacy aspects and was therefore assessed. In most cases, the occurrence of data upload was not mentioned and thus it could not be determined whether data was uploaded or not; this was the case in 63.7% (74/116) of the selected tools (Table 2). However, in the case of 10.3% (12/116) of the tools, it was mentioned that data was only stored locally on the users device and was thus not uploaded. User data upload was specifically mentioned in 25.9% (30/116) of the tools, though it was still unclear what exactly happened with the uploaded data. These results indicate that information regarding data upload, including both the occurrence of upload and the use/storage of data, is largely missing.

For the tools designed to be used by non-health care professionals, we assessed whether a health care professional was (supposed to be) involved in *obtaining* or *using* the tool. For *obtaining* a tool, this was not the case for most tools; only 13.8% (16/116) were intended to be obtained with the help of a health care professional, while 70% (80/116) were not intended to be (Table 2). For 3.4% (4/116) of the tools, we could not determine whether or not this intention was present making assessment impossible. Roughly, the same could be seen when it came to whether or not a health care professional was intended to be involved during *use* of a tool. The majority, 68.1% (79/116), was not intended to be used with the help or involvement of a health care professional. Only 14.6% (17/116) was intended to be used in that way.

In addition, we assessed whether the tools could (accidentally) partially replace a health care professional, which is sometimes the intended aim of a tool to reduce health care costs. These were included in the benefits described above. However, (accidental) replacement of a health care professional is also a possible risk. For only 5.2% (6/116) of the apps, we identified that they could possibly (partially) replace a health care professional, while 93.1% (108/116) could not (Table 2). These results indicate that replacement of health care professionals is not a large concern considering apps related to medication use.

Finally, we assessed if (incorrect) use of the tool or a possible error in the tool could lead to incorrect decisions with a large impact on the users’ health (Table 3). In the case of (incorrect) use there was a large number of tools that (if used incorrectly) could have a negative impact on the users’ health; however, in 61.2% (71/116) the chance of this actually happening was unrealistic. In only 2.6% (3/116) the chance was deemed realistic. For 34.5% (40/116) of the tools, there was no chance that (incorrect) use of the tool could have a large impact on the users’ health. The chance of a possible error in the tools that could lead to decisions with a large impact on users’ health was higher. This chance was deemed realistic in 6.0% (7/116) of the tools and the chance was present but unrealistic in 88.8% (103/116) of the tools. In only 2.6% (3/116) of the tools this chance was not present at all. In summary, these results indicate that most of the apps and tools related to medication have low health risks or patient safety risks; however, a small subset of the apps could be identified as having more possible health risks involved. For a complete overview of the results considering potential risks, see Table 2 and Table 3.

**Table 2:** Assessment of possible risks of apps and other e-tools for medication use, based on tool characteristics; data is presented as percentage of total number of tools investigated (N=116).

Coding	Yes	No	Not assessable	Not applicable
	n (%)	n (%)	n (%)	n (%)
Data upload	30 (25.9)	12 (10.3)	72 (62.1)	0 (0)
Health care professional involved in obtaining e-tool	16 (13.8)	80 (69)	4 (3.4)	16 (13.8)
Health care professional involved in using e-tool	17 (14.6)	79 (68.1)	4 (3.4)	16 (13.8)
Accessible to non-health care professionals if type II medical device	8 (6.9)	0 (0)	1 (0.9)	108 (93.1)
Replaces health care professional	6 (5.2)	108 (93.1)	2 (1.7)	0 (0)

**Table 3:** Assessment of possible risks of apps and other e-tools for medication use, when errors (in use or the tool) are present; data is presented as percentage of total number of tools investigated (N=116).

Coding	Yes, realistic	Yes, but not realistic	No	Not assessable
	n (%)	n (%)	n (%)	n (%)
Can erroneous use of the e-tool pose a health risk and is chance realistic ?	3 (2.9)	71 (61.2)	40 (34.5)	2 (1.7)
Can an error in the e-tool pose a health risk and is chance realistic?	7 (6.0)	103 (88.8)	3 (2.6)	2 (1.7)

### Online Questionnaires Among Users

#### Participants

After the first DVN newsletter, 201 persons with diabetes responded. A reminder invitation was placed on the website of the organization (DVN). After this reminder, 75 additional people with diabetes responded. In total, 276 people with diabetes responded. However, 36 respondents only answered the first question of the questionnaire, “Do you use apps for regulating blood glucose levels?”, but did not complete the rest of the questionnaire and were thus excluded from the analysis. This resulted in 240 respondents included in the final analysis, which is 0.5% of possible invitees (about 49,000 members of DVN).

#### Characteristics of Respondents

Questions regarding demographic characteristics of respondents were optional. For 208 respondents age category is known. Age distribution of these respondents is as follows: <20 years: n=13, 5%; 20-40 years: n=31, 13%; 41-65 years: n=100, 42%; 66-75 years: n=46, 19%; >75 years: n=18, 8%; no answer: n=32, 13%. Only a small number of respondents indicated to not use any type of medication for diabetes (n=5, 2%).

#### Use of Apps and Characteristics of Apps

Around one third of the respondents used apps for regulating their blood glucose levels, whereas almost two thirds indicated not to use apps for this purpose. A small minority of respondents indicated that they have used apps before, but stopped using them and some respondents downloaded an app for regulating blood glucose levels but never used them (Table 4). These results show that of the respondents who have ever downloaded app(s) for regulating blood glucose levels, 17.7% (20/113) never used the app and 15.0% (17/113) stopped using them after short or long term use. Indicating, that around two third of the respondents that have downloaded app(s) is using them at the moment.

Of the 147 respondents that never used apps, 23.8% (35/147) indicated that an important reason not to use apps is that they don’t know how it works and 22.4% (33/147) indicated that they do not know if the apps are reliable (Multimedia Appendix 1 supplementary data E Fig E1). In addition, 51 respondents indicated that they have “other” reasons for not using apps. The most often mentioned other reason was that they did not know apps existed (n=20). Of the respondents that indicated not to use apps anymore (n=17), 10 respondents indicated that the main reason was that the apps did not function well and took too much time.

**Figure 4:**
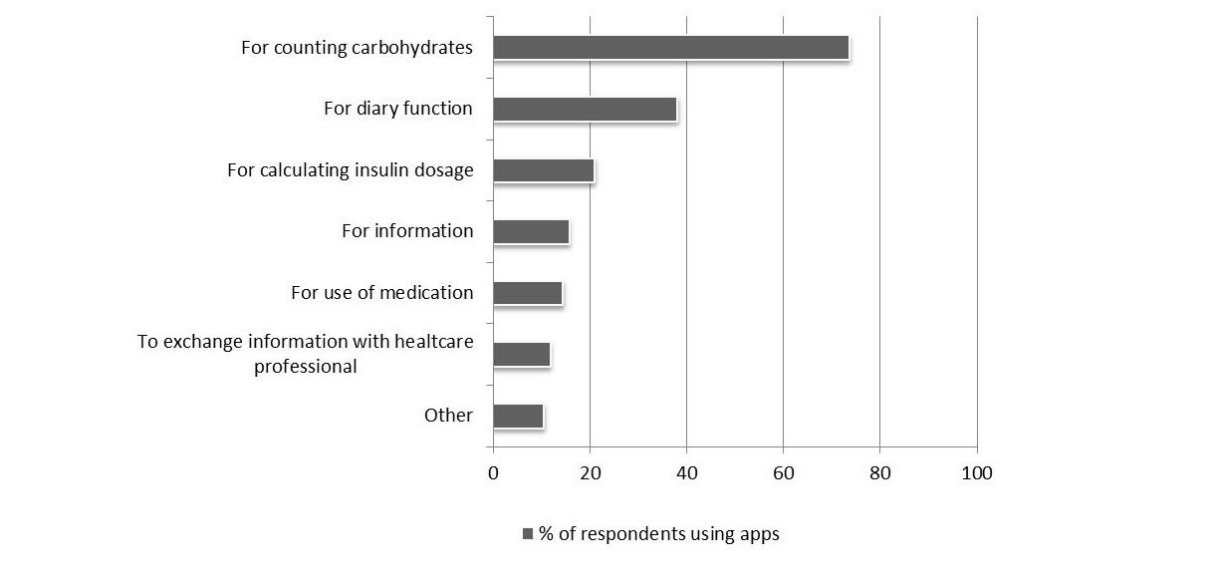
Types of apps used by people with diabetes. Data is presented as percentage of respondents that use apps (n = 76). Multiple answers were allowed. Two respondents using apps did not answer this question (3 %).

Most respondents reported to use an app for counting carbohydrates (Figure 4). In addition, apps with a diary function and insulin dosage calculators were often used (Figure 4).

#### Selection of Apps

**Figure 5:**
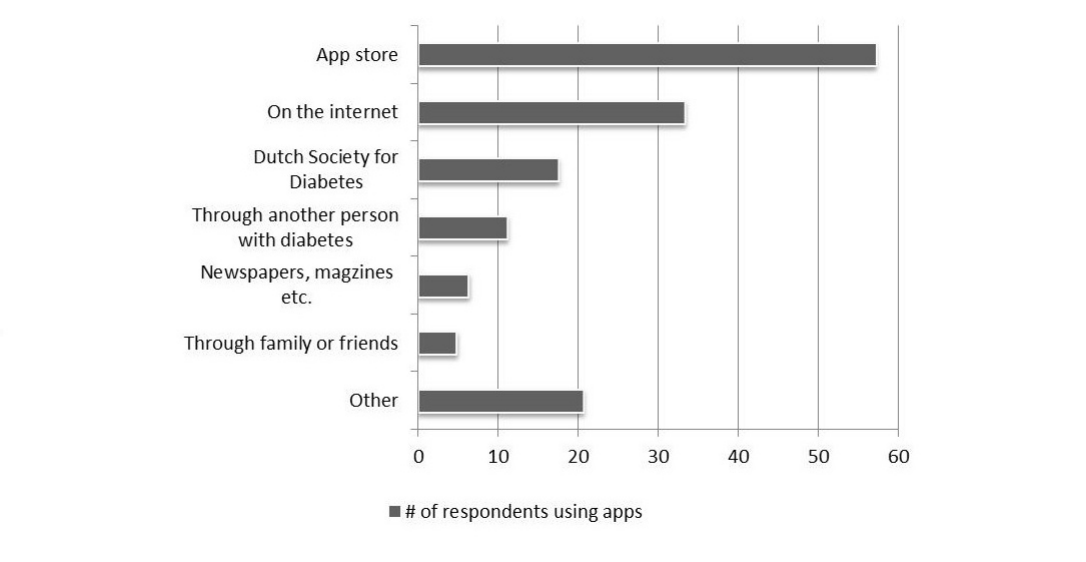
Popularity of different sources for obtaining aps. Data is presented as percentage of respondents using apps and that answered this question (n =63 out of 76 users). Multiple answers were allowed.

The app store is the most popular place to find (Figure 5). Thirteen respondents indicated to use other sources for finding apps. This included health care professionals (n=6) and building their own app (n=2). In addition, we asked respondents how they knew the apps that they used were reliable (68 respondents provided 80 answers). Most frequently, respondents indicated that they did not know if the apps were reliable (n=16). Fourteen respondents indicated that they check with other sources. Other responses included: assumed that the apps are reliable (n=8), apps are advised by or discussed with a health care professional (n=7), and comparison with previous measurements (n=7). Apps that perform calculations for diagnostic or therapeutic purposes are medical devices for which a CE mark is required. The majority of the respondents indicated that they did not know if a CE mark was present (75%, 21/28). Only 3 respondents indicated that a CE mark was present (11%) and 4 respondents indicated that no CE mark was present (14%) (Multimedia Appendix 1 supplementary data Fig E2).

**Table 4:** Use of apps for regulating blood glucose levels by persons with diabetes; data are presented as percentage of respondents (N=240).

Use of apps for regulating blood glucose levels	
Answer options	Respondents, n (%)
Yes	76 (31.7)
Not anymore	17 (7.1)
No, downloaded but never used	20 (8.3)
No	127 (52.9)

#### Risks and Benefits

Respondents using apps (n=76) were asked to name advantages of doing so in an open question and subsequently were asked how they experienced a set of four predefined benefits. Sixty three persons provided 82 responses to the open question. The advantages mentioned most often are: apps are convenient to use (n=26) and information quickly and simply available (n=25). Additional advantages that were mentioned are: better insight into your own health (n=16) and improves healthy lifestyle (n=8).

Respondents were asked how frequent they experience a set of 5 benefits possibly associated with the use of apps. A scale of 1-5 was used with 1 representing never and 5 representing very often. For the predefined set of 5 benefits, the benefits “information quickly available”, “improves my health”, and “improves my independency” are experienced frequently by the respondents (60 out of 76 users of apps). These benefits are experienced often or very often by 78%, 71%, and 58% of the respondents, respectively (scale 4 and 5) (Table 5). The benefit “helps with correct use of medication” is experienced not at all or rarely by two third of the respondents (65%, scale 1 and 2). Of the respondents using apps, 16 respondents (21%) did not answer this question.

Respondents who were using apps were asked to name disadvantages of using apps and subsequently, were asked how they experienced a set of 5 predefined risks.

Of the respondents using apps (n=76), 59 persons provided a total of 67 responses of which 25 respondents indicated not to experience any disadvantages. The disadvantages mentioned most often are: “apps are incomplete (information and functionalities)” (n=21) and “usage takes too much time” (n=8). Of the set of 5 predefined risks, only very few respondents indicated to experience these risks, see Table 5. Risks experienced the most frequent are problems or doubts concerning reliability of calculations and information (Table 6). Respondents were asked how frequent they experience a set of 5 risks possibly associated with the use of apps. A scale of 1-5 was used with 1 representing never and 5 representing very often. Data are expressed as percentage of respondents using apps and that answered this question (n=58). Of the respondents using apps, 18 respondents (23.7%) did not answer this question. For reliability of calculations, these risks are experienced sometimes, often or very often by 22% (13/58) of the respondents and by 19% (11/58) of respondents for reliability of information (scale 3, 4 and 5, Table 5).

**Table 5:** Benefits experienced by use of apps for regulation of blood glucose levels. Experiencing benefits 1.

Experiencing benefits of app use (n=76)					
	1	2	3	4	5
Information quickly available, n(%)	5 (8)	2 (3)	6 (10)	16 (27)	31 (52)
Helps with correct use of medication, n(%)	36 (60)	3 (5)	4 (7)	7 (12)	10 (17)
Helps with correctly setting insulin dosage, n(%)	21 (35)	1 (2)	10 (17)	11 (18)	17 (28)
Improves my health	6 (10)	1 (2)	10 (17)	19 (32)	24 (40)
Improves my independency	12 (20)	1 (2)	12 (20)	11 (18)	24 (40)

^1^Data are expressed as percentage of respondents using apps and that answered this question (n=60), Scale 1-5: 1=never or not applicable, 5=very often.

**Table 6:** Risks experienced by use of apps for regulation of blood glucose levels 1.

Experiencing risks of app use^1^n(%)					
	1	2	3	4	5
Problems or doubts concerning privacy when data is entered	46 (79)	7 (12)	4 (7)	0 (0)	1 (2)
Problems or doubts concerning reliability of calculations	38 (66)	7 (12)	11 (19)	0 (0)	2 (3)
Problems or doubts concerning reliability of information	34 (59)	9 (2)	9 (16)	0 (0)	2 (3)
Problems with availability of the app	50 (86)	4 (7)	2 (3)	1 (2)	1 (2)
Complicated use of the app	46 (79)	7 (12)	3 (5)	1 (2)	1 (2)

^1^Scale 1-5: 1=never or not applicable, 5=very often.

Respondents were also asked how they manage the risks and disadvantages of using apps, 45 respondents provided 48 answers. Sixteen respondents indicated that they use another source or look for another source. Nine respondents indicated that they do not experience disadvantages. Other responses were: accept and deal with the disadvantages (n=6), and use my own indication and/or guess (n=5).

## Discussion

### Principal Findings

The aim of the present study was to gain more insight into the characteristics, possible risks, and possible benefits of health apps and e-tools related to medication use. Therefore, an inventory of apps and e-tools was made and subsequently users’ experiences were investigated by an online questionnaire. The present study shows that for apps and e-tools related to medication use a small subset of tools might involve relatively high risks. For the large group of nonmedical devices apps the risks are lower, but risks lie in the enormous availability and low levels of regulation. Results of the online questionnaire show that respondents using apps for regulation of blood glucose levels experience many benefits and little risks. In addition, both users and nonusers indicated that overall quality of apps (ease of use, completeness, and good functionalities) is an issue. Nonusers indicated that they might consider using apps in the future if they receive evidence regarding the reliability of apps and better instructions on how to use them.

### Search Strategy for Apps and Limitations of the Study

When performing the search for apps related to medication use, we encountered several challenges. First, we used two different app stores for the present search (iTunes and Google Play store). These app stores had different search possibilities and there is no information present on how search results are provided to the consumer/researcher. Search results changed frequently, probably partially due to a change in the availability of the apps. New apps are appearing every day and apps are disappearing as well. As a consequence, the selection of apps and information related to these apps is a snapshot of one moment in time. In the subsequently performed online questionnaire, we did not focus on specific apps, but rather on one type of app users, and thus, the large dynamic content of the app stores is less an issue.

The possibilities for using search strings in the app stores are very limited. App stores are, understandably, not designed for scientific research. This limitation led to a high number of hits and enormous amounts of irrelevant apps, which made using cut-offs necessary for this study (see Methods section). Similar problems were identified in previous studies by Aungst et al as well [26-28]. Users will not perform systematic searches when looking for apps; however, they most likely encounter the same amount of irrelevant apps that they have to search through to find what they are looking for. Results of the online questionnaires among users show that the respondents mainly use the app stores for finding apps, but other sources are used as well.

Apart from the limitations of the search possibilities, other limitations of this study include the assessment of the possible risks and benefits. To increase objectivity these assessments were performed by two independent researchers according to fixed criteria and we used an online questionnaire among users of one type of frequently downloaded apps (for persons with diabetes). However, only a small subset of users responded to the online questionnaires and therefore, the complete prevalence of benefits and risks cannot be determined. In addition, a selection bias is likely present. For example, age distribution of the respondents is somewhat younger compared to the age distribution of people with diabetes in the Netherlands [26]. Therefore, this study group cannot be seen as representable for the entire population determined. However, this study provides a good insight into possible and experienced benefits and risks and indicates where improvements need to be made.

### Characteristics and Use of Apps

In our selection of tools related to medication use, the most prevalent functionalities were medication adherence, monitoring of effects/adverse reactions, and providing information. Most of the tools aid in daily use of medicines: reminders with alarms for taking medication (medication adherence) and diary functions to monitor effects/adverse reactions. Hence, these tools do not provide new functionalities compared to existing technology such as an alarm clock, a written diary, and a textbook or brochure for information. However, they make the use of these tools easier by enabling these functions on the patients’ mobile phone allowing them to have these tools with them wherever they go. Results of the online questionnaires indicate that “having information quick and conveniently available” is indeed one of the most experienced benefits of using apps by persons with diabetes (78.3%).

The functionalities identified in this study are comparable to previously identified functionalities [2,27]. A study among consumer health apps available in the iTunes app store also identified a large set of apps with the “inform” functionality. In addition, “record” and “remind/alert” were functionalities identified in a substantial amount of apps [2]. The majority of the apps and e-tools investigated were aimed at patients, only a minority was aimed solely at health care professionals. However, some of the most downloaded apps were aimed at health care professionals (eg, “anesthesiologist”). Similar to a previous study by IMS [2], we observed that the majority of apps are being downloaded sparsely. Only a few apps are downloaded very frequently. One of the most downloaded apps in our selection aimed at patients was “glucose buddy”, a diabetes management app. It is important to note that the data on downloads was only available for apps in the Google Play Store. In addition, it is unclear how the number of downloads represents actual usages. Results of the online questionnaire show that around two third of the respondents who have downloaded app(s) are using them at the moment. This indicates that download numbers are related to actual use of the apps to a reasonable extend.

In summary, the inventory showed that functionalities of apps for medication use are not really new compared to previously available resources. Results of the online questionnaires show that users indeed mainly use the apps because they are convenient and download numbers relate to actual use of these apps to a reasonable extend.

### Possible Benefits

To identify the possible benefits of apps and e-tools, for the apps identified in the inventory, we coded different aspects that are generally named as benefits of eHealth, such as lowering health costs, improving health care, enhancing patient self-management and self-reliance [23-25]. Of these potential benefits, “improving health” and “enhancing patient self-reliance” were most prevalent among selection of apps and e-tools for medication use in the inventory. Results of the online questionnaire show that among users of apps “improving health” is experienced often or very often by more than two thirds of the respondents. Enhancing self-reliance is experienced often or very often by around half of the responding persons with diabetes. These findings indicate that the intended benefits of the apps for medication use (as identified in the inventory) are also experienced by a substantial part of the users.

Research providing evidence-based information regarding actual benefits of apps is limited [15,29]. Therefore, it is important that the use of apps and e-tools is investigated for their effectiveness at having the desired benefits (eg, improving health, lowering health costs, enhancing patient self-management etc). Interestingly, a recent study indicates that the effectiveness of a diabetes management app to “improve health” is largely dependent on patient willingness to use the app and satisfaction with the app [30], indicating the need to include these aspects in studies investigating benefits and effectiveness of e-tools. This is in line with our study, which shows that of the people willing to use the app and doing so, many feel that the app(s) improve their health.

In summary, based on the inventory and experiences of users, our study suggests that important benefits such as “improving health” and “improving self-reliance” can be gained from the use of apps.

### Possible Risks and Disadvantages

We assessed possible risks of apps and e-tools related to medication use in the inventory and asked respondents using apps to their experiences. Some of these risks investigated in the present study have previously been identified as possible risks of mobile medical apps [31].

Overall, for only a small subset of the apps in the inventory, relatively high potential risks were observed (medical device and available without involvement of a health care professional). These are mainly related to apps where errors in the apps or erroneous use may lead to substantial health risks, such as apps for calculating insulin dosages for people with diabetes. The results of the online questionnaire among persons with diabetes show that, overall, respondents that use apps for regulating blood glucose levels experience little risks and disadvantages of app use.

Problems with or doubts on reliability of calculations are experienced sometimes to very often by about one fifth of the persons with diabetes using apps. This indicates that for most responding users this is not much of an issue. In addition, respondents indicated that they have several ways to manage these risks (such as checking with multiple sources, etc). Therefore, it is not known whether problems actually have serious negative consequences. Previous studies have investigated the accuracy of medical calculation apps for medical professionals [32] and insulin calculators [18]. Bierbrier et al reported that the calculations in the medical calculation apps, including dosage calculations, were accurate and reliable with a few exceptions (mainly scorings related to liver disease) [32]. In contrast, systematic assessment of accuracy and clinical suitability of apps calculating insulin dose showed that only 1 out of 46 apps was issue-free [18]. In the present study, incompleteness of apps (regarding information and functions) and poor ease of use were the most frequent mentioned disadvantages of use by the respondents. Hence, there is clearly room for improvement regarding the quality of apps when information, functionalities, and ease of use are considered.

For the majority of apps in the inventory, it is unclear whether and how personal data is uploaded, which is relevant for patient privacy aspects. Developers should improve their provision of information regarding data upload and storage. Interestingly, results from the online questionnaires indicate that the risks with privacy are not recognized or considered relevant by most of the respondents using apps. Only a small minority of the users mentioned this as a risk of using apps, a reason not to use apps, or experienced issues with privacy sometimes.

In summary, the inventory indicated that only a few apps have potentially high risks and for many of the apps it is unclear whether and how personal data are stored. Online questionnaire among users of apps for blood glucose regulation indicate that they experience problems or doubts considering reliability and privacy very sparsely. In contrast, they do mention to experience disadvantages of use due to incomplete apps and apps with poor ease of use.

### Selection of Apps and Reasons Not to Use Apps

In the past years, the availability of the apps and e-tools has increased enormously. Considering this enormous availability of apps and low levels of regulation (only a small portion of the apps in the inventory could be classified as a medical device (according to EU regulations [7,22]), patients and health care professionals might have little indications as to which e-tools are reliable. In the online questionnaire, users of apps for regulating blood glucose levels were asked how they knew if the apps that they used were reliable. Most respondents indicated that they did not know this. This problem has previously been described as well by Velsen et al [33] and by Lewis et al [31]. In this latter publication, a scheme for categorizing apps according to associated risks was described together with suggestions for assessment of these risks by, for example, peer review and/or best practice guidelines [31]. Among the respondents of the online questionnaire not using apps, “advice on which apps are reliable” was the most often mentioned requirement for them to start using apps, indicating that among potential users there is a need for better instruction or visibility of reliable apps. Recently, there have been several initiatives to make choosing reliable apps easier for patients and health care professionals by enabling peer-review. These initiatives are mainly websites where health care professionals and patients review health apps, for example: the online health apps library by the National Health Service, UK [34]; online database iMedicalApps where health care professionals review apps for health care professionals and patients [35]; online database of medical apps by a Dutch association for health care professionals [36]; appill by everhywhereIM, a database of medical apps for health care professionals and reviews by health care professionals [37]. However, it will be impossible to review and/or regulate all available e-tools due to the enormous availability and dynamic nature. As stated by Boulos et al, the best first line of defense is to educate consumers, patients and health care professionals about the risks and the proper caution that is required when using apps [15].

Interestingly, results of the online questionnaires indicate that the main reasons for not using apps are unfamiliarity with the existence of apps or with how the apps work and concerns regarding the reliability of apps. Considering the possible benefits that can be obtained from app use, improving quality of apps, awareness of apps, and how to use them, might lead to better use of eHealth benefits. It appears that there is a role for developers of apps, government agencies, health care professionals, competent authorities, patient organizations and/or other stakeholders in developing high quality apps and providing advice and instruction to patients and the general public regarding proper use of apps.

### Conclusions

In summary, the present study shows that for apps and e-tools related to medication use a small subset of tools might involve relatively high risks (medical devices-class II and used by patients without involvement of health care professionals). For the large group of apps that are nonmedical devices, risks are lower, but risks lie in the enormous availability and low levels of regulation. In addition, both users and nonusers indicated that overall quality of apps (ease of use, completeness, good functionalities) is an issue. Considering that important benefits (eg, improving health and self-reliance) are experienced by many of the respondents using apps for regulating blood glucose levels, improving reliability and quality of apps is likely to have many profits. In addition, creating better awareness regarding the existence and how to use apps properly will likely improve proper use by more people, enhancing the profits of these tools.

## Appendix

Multimedia Appendix 1 Supplementary data.

## Abbreviations

FDA: Food and Drug Administration
DVN: Dutch Diabetes Association
