# Supplementary material for: Characterization of Apps and Other e-Tools for Medication Use: Insights Into Possible Benefits and Risks
Source: JMIR Mhealth Uhealth. 2016 Apr 6;4(2):e34. doi: 10.2196/mhealth.4149 (PMC4838755; doi:10.2196/mhealth.4149)
Supplement: Multimedia Appendix 1 [file mhealth_v4i2e34_app1.pdf]

Supplementary data

Supplementary data A: Additional information search strategy inventory of apps for medication use

Translation of Dutch search terms and websites searched

English	Dutch
Medicine	Medicijn
Drugs	Geneesmiddelen
Diabetes	Diabetes
Asthma	Astma
Breast cancer	Borstkanker
Prostate cancer	Prostaat kanker
Cardiovascular diseases	Hart- en vaatziekten
ADHD	ADHD

Websites searched:

www.npcf.nl

www.zelfmanagment.com

www.skipr.nl

www.nictiz.nl

www.knmp.nl

www.ehealth.nl

www.medicalfacts.nl

www.dvn.nl

www.longfonds.nl

www.nfk.nl

www.hartenvaatgroep.nl

Supplementary data B: Additional information coding of selected apps for medication use

Explanation and criteria of codes

All apps were individually assessed for all the codes described below. For some codes, groups of apps are described for which a coding was applicable. For example, when certain functionality was always coded with a certain benefit, but apps were always assessed individually. During the coding process 14 apps were excluded from the original selection because too little information was provided in the app stores, resulting in a total of 116 coded tools. Codes were performed by L. van Kerkhof (PhD, Biomedical Sciences) and C. de Jong (Msc, European Studies) and supervised by I. Hegger (PharmD).

Characteristics

Functionalities: Codes were based on pilot searches and pilot codes.

Users: Codes were based on Nictiz Whitepaper' Orde in the world of eHealth' [18]. Tools were coded with 'patient and healthcare professional' when the interaction between both was clearly an intended use of the tool. For example, when it was clearly described that the tool should be used to send information to you healthcare professional. If this was not clearly stated tools were coded as 'intended user – patient'

Technical domain: Codes were based on Nictiz Whitepaper 'Order in the world of eHealth' [18].

Downloads: Downloads were obtained from the information provided with the apps from the Google Play store. For apps from the iTunes Store download numbers were not available.

Benefits

Self-reliance: Does the tool potentially enhance a patient's self-reliance? Apps that are able to make a patient more independent of a health care professional or others. Apps with the functionality of 'improving therapy adherence' and 'monitoring effects or side effects' were coded with 'self-reliance –yes'.

Improving health: Does the tool potentially improve health? Examples of apps coded with 'improve health - yes' are apps with the functionality of 'improving therapy adherence' or 'monitoring effects or side effects'. Apps that only provide information are not considered to improve health in this question, nor are dosage calculators for healthcare professionals.

Lowering health care costs: Has the tool the potential to lower health care costs? Tools coded with functionality 'improving therapy adherence' 'or the benefit of 'enhancing self-reliance' are considered to potentially lower health care costs.

Improving self-management: Does the tool potentially improve self-management? Self-management was considered when the tool helped users actively participate in the decision-making of their own therapy. This mainly included, but not exclusively, apps with the functionality of 'monitoring effects or side effects'

Risks

Medical device: Coding of medical device and class was based on EU regulations [7] and the Nictiz Whitepaper: 'Medical apps, is CE-mark required?' [23]. Apps aimed at monitoring diabetes were only coded as class II medical device when it was clearly stated that calculations (medication dose) were made by the app.

Data upload: Codes regarding data upload were coded with 'no' or 'yes' if this was specifically mentioned in the tools description. In addition, 'yes' was coded when it was clear that data was transferred when using the tool in a normal way.

Healthcare professional involved in obtaining the tool or during use of the tool: Tools were coded with 'yes' on this question when it was clearly stated that a healthcare professional should be involved or when a code was required to obtain or use the tool.

Replacement of health care professional: Is the goal of the tool to (partially) replace a health care professional or can this happen by accident? Tools were coded with 'yes' on this question when tools could replace healthcare professionals. Specifically, tools with the functionality of only 'providing information' were not coded with 'yes'.

Can incorrect use or incorrect design of the tool result into decisions with a large impact on the users health? Four different codes were used for this question: ‘yes and realistic’, ‘yes, but not realistic’, ‘no’ and ‘not assessable’. The first two codes were used to distinguish between theoretical risks that might occur and an estimation of these risks actually happening. For example, when a healthcare professional is involved risks were considered less likely to happen considering the education of healthcare professionals, while when only patients are involved risks might be more likely to occur.

Supplementary data C: Online questionnaires

* = obligated question

Online questionnaire A (start for all persons with diabetes)

Topic	*Question*	*Answer options*	*Additional information (not a visible part of the questionnaire)*
Use of apps	Do you use apps for regulating blood glucose levels? *	- Yes<br>- No, I never used them<br>- No, I tried them but I stopped using them<br>- No, I downloaded them, but never used them	*Multiple choice, only 1 answer possible*<br><br>*If ‘no, I never used them’ or ‘no, I downloaded tem, but never used them’ go to questionnaire B*<br>*If ‘no, I stopped using them’ go to questionnaire C*
	What are the names of the apps that you use for regulating blood glucose levels?		*Open question*
	What type of apps do you use for regulating your blood glucose levels?	- For counting carbohydrates<br>- For diary function<br>- For information<br>- For calculating insulin dosage<br>- To exchange information with healthcare professional<br>- For use of medication<br>- Other, namely:	*Multiple choice, multiple answers allowed. Includes open question.*
	Does the app for calculating	- Yes	*Multiple choice, only 1*

	insulin dosages that you use have CE-Mark?	- No<br>- I don't know<br>- I don't use these apps	*answer possible*<br><br>*Picture of a CE-Mark is shown with the question.*
	How did you find the apps that you use for work?	- Search in the app store<br>- Newspapers, magazines etc.<br>- Dutch Diabetes Society (DVN)<br>- Through another person with diabetes<br>- Through family or friends<br>- On the internet<br>- Other, namely:	*Multiple choice, multiple answers allowed. Includes open question.*
	How do you know if the apps you use are reliable?		*Open question*
Benefits and risks of apps	What are the benefits you experience by the use of apps for regulating blood glucose levels?		*Open question*
	To what extend do you experience the following benefits by the apps that you use?	Scale of 1-5 per answer<br>- Information quickly available<br>- Helps with correct use of medication<br>- Helps with correctly setting insulin dosage<br>- Improves my health<br>- Improves my independency	*For all options a scale from 1-5 needs to be completed. The following meaning of the scale is presented with the question: Scale 1-5, with 1 representing I don't experience this at all or not applicable, to 5 representing I experience this very often.*
	What are disadvantages that you experience by using apps for regulating blood glucose levels?		*Open question*
	To what extend do you experience the following risks by the apps that you use?	Scale of 1-5 per answer<br>- Problems or doubts concerning privacy when data is entered<br>- Problems or doubts concerning the reliability of calculations<br>- Problems or doubts concerning the reliability of information	*For all options a scale from 1-5 needs to be completed. The following meaning of the scale is presented with the question: Scale 1-5, with 1 representing I don't experience this at all or not applicable, to 5 representing I experience this very often.*

		- Problems with the availability of the app<br>- Complicated use of the app	
	How do you manage these risks?		*Open question*
**From questionnaire A to final questions for all**			
**Questionnaire B** (only for respondents not using apps)			
Use of apps	What are the reasons that you don’t use apps for regulating blood glucose levels?	- I don’t have the required equipment (tablet of smartphone)<br>- I am not interested<br>- I don’t know how it works<br>- I don’t know if the apps are reliable<br>- Other, namely:	*Multiple choice, multiple answers allowed. Includes open question.*
**From questionnaire B to final questions for all**			
**Questionnaire C** (only for respondents that stopped using apps)			
Use of apps	What are the reasons that you stopped using apps for regulating blood glucose levels?		*Open question*
**Final questions for all respondents**			
Personal information	What is your age category	- < 30<br>- 30 – 40<br>- 40 – 50<br>- 50 -60<br>- >60	*Multiple choice, only 1 answer possible*
	What type of medication do you use?	- Insulin<br>- GLP-1 analog<br>- Pills<br>- Insulin and pills<br>- No medication	*Multiple choice, multiple answers allowed. Includes open question*
	Do you have any additional remarks?		Open question

Supplementary data D: Additional results inventory apps and e-tools

Table D1: Characteristics of tools (functionality, technical domain, and intended user). Data is presented as absolute numbers (n) and as percentage of total number of tools (%, N=116).

Main characteristics

Intended functions	**N**	**%**
Therapy adherence	43	37.1
Monitor effects/adverse effects	43	37.1
Drug interaction monitoring	13	11.2
information/education	61	52.6
Choice of medicine/dose	23	19.8
Research	4	3.4
Diagnostic measuring	1	0.9
Self-preparation of medicine	1	0.9
News	9	7.8
Other	7	6.0

Technical class	**N**	**%**
App	101	87.1
Internet	14	12.1
Measurement tool	6	5.2
Video	1	0.9
Electronic health record	1	0.9
Other	4	3.4

Intended user	**N**	**%**
Healthcare professionals	15	12.9
Healthcare professionals among each other	1	0.9
Patient	69	59.5
Patients among each other	0	0.0
Healthcare professional and patient	27	23.3
Patients and others	3	2.6
Healthcare professional and other	1	0.9

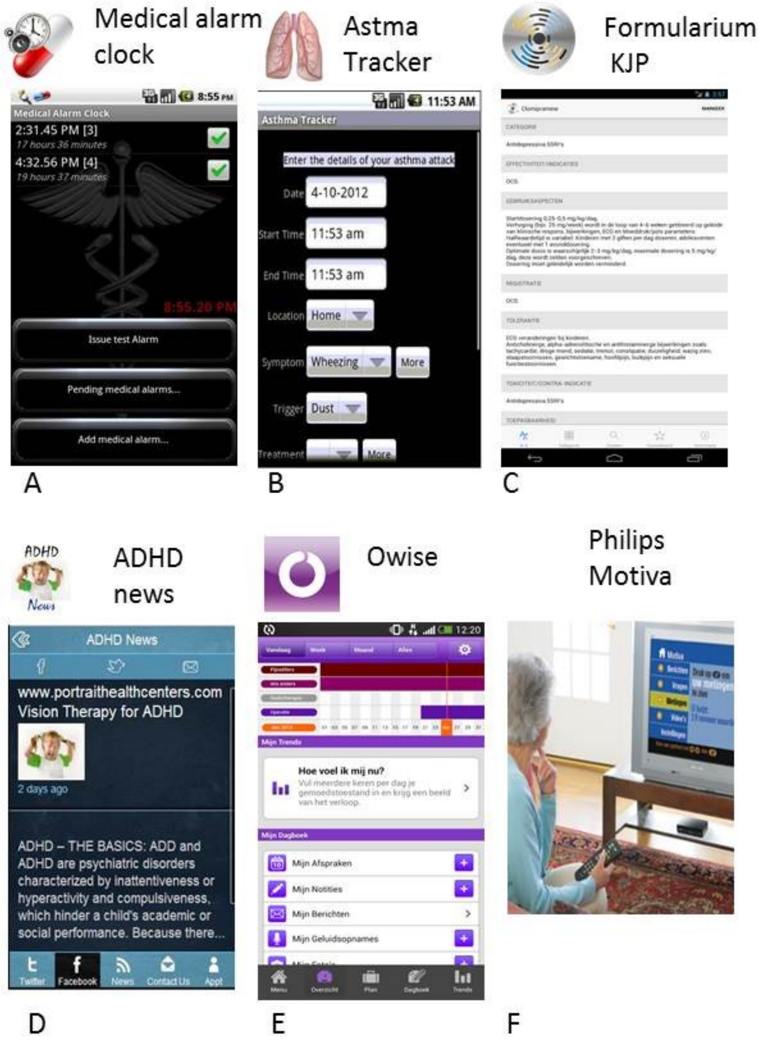

Fig. D1: Examples of apps included in the inventory. Screenshots of the apps were taken directly from the app stores or websites of e-tools (not from the tools). Examples of app with different functionalities are given: therapy adherence (A), monitoring effects/adverse effects (B), choice of medicine/dose (C), information/education and news (D), research (E), and diagnostic measuring (F).

Supplementary data E: Additional data online questionnaire

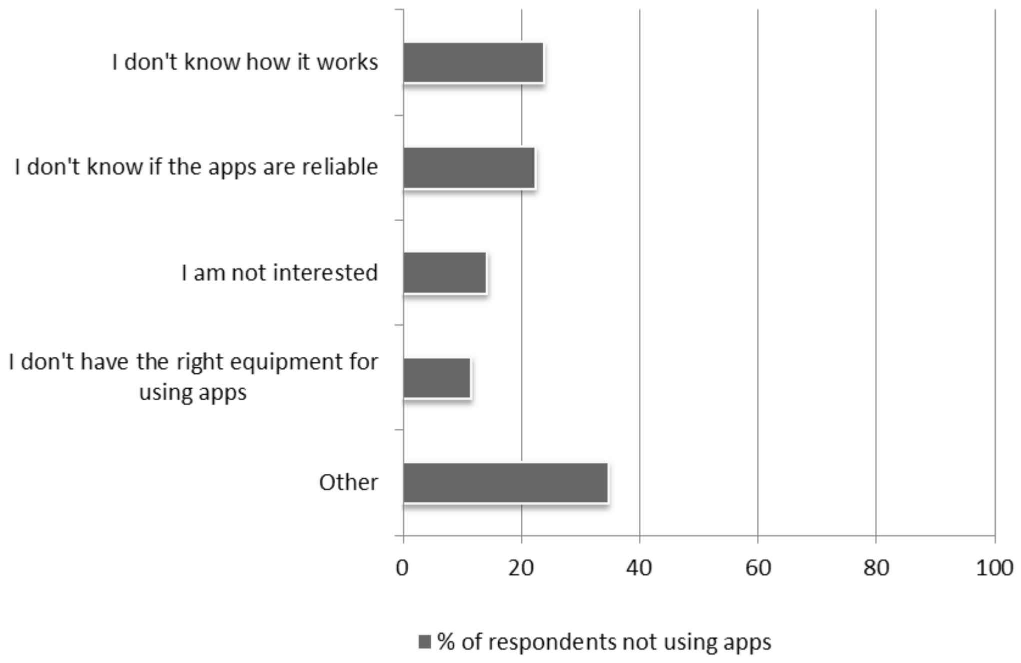

Fig. E1: Reasons for not using apps. Data is presented as percentage of respondents not using apps (n = 147).

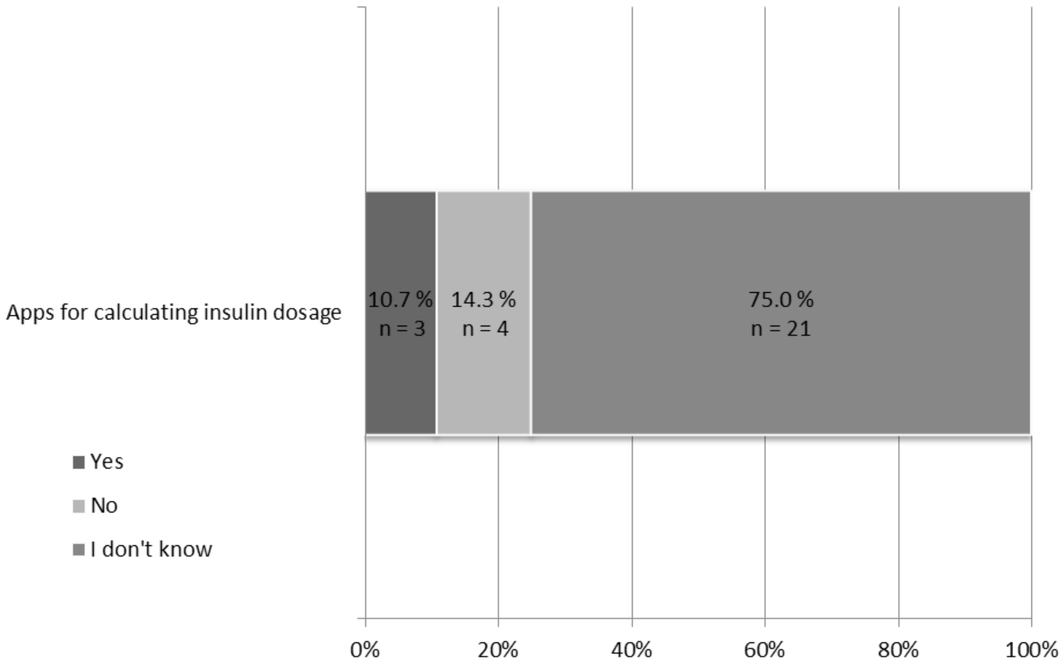

Fig. E2: Presence of a CE-mark on the apps for dosage calculators. Data is presented as percentage of users that indicated to use these apps and have answered this question (n = 28). This question was not answered by 9 respondents and 39 respondents indicated not to use these apps.
